# The Assessment of the Severity of COVID-19-Related Anxiety Symptoms in Participants of the University of the Third Age in Poland: A Cross-Sectional Study among Internet Survey Respondents

**DOI:** 10.3390/jcm10173862

**Published:** 2021-08-27

**Authors:** Mateusz Cybulski, Urszula Cwalina, Dorota Sadowska, Elżbieta Krajewska-Kułak

**Affiliations:** 1 Department of Integrated Medical Care, Faculty of Health Sciences, Medical University of Białystok, M. Skłodowskiej-Curie 7A str., 15-096 Białystok, Poland; elzbieta.krajewska-kulak@umb.edu.pl; 2 Department of Medical Statistics and Health Informatics, Faculty of Health Sciences, Medical University of Białystok, Szpitalna 37 str., 15-295 Białystok, Poland; urszula.cwalina@umb.edu.pl; 3 Department of Endocrinology, Diabetology and Internal Medicine, Faculty of Medicine, Medical University of Białystok, M. Skłodowskiej-Curie 24A str., 15-276 Białystok, Poland; dorotasad@gmail.com

**Keywords:** anxiety, COVID-19, fear, general anxiety disorder-7 (GAD-7), older adults, SARS-CoV-2, short health anxiety inventory (SHAI), state-trait anxiety inventory (STAI)

## Abstract

Introduction: Fear of infection with SARS-CoV-2 has become widespread. All over the world, since the very beginning of the pandemic, older adults have been considered one of the groups at highest risk of SARS-CoV-2 infection and death due to COVID-19. The aim of the study was to evaluate the severity of anxiety symptoms related to COVID-19 in the older adults who are participants of the Universities of the Third Age in Poland. Material and methods: The study included participants of the University of the Third Age in Poland. A total of 296 persons were enrolled, including 258 women and 38 men. The study was a diagnostic survey, conducted with the use of the following validated psychometric scales: General Anxiety Disorder-7 (GAD-7), Short Health Anxiety Inventory (SHAI), and State-Trait Anxiety Inventory (STAI). Results: In two scales (STAI and SHAI), the mean scores demonstrated mild symptoms indicative of anxiety disorders in the older respondents. Women and men did differ significantly in terms of the scores obtained in STAI X-1 and STAI X-2. Single respondents differed significantly from divorced ones in terms of STAI X-1 scores. Moreover, widows/widowers differed significantly from divorced ones in terms of STAI X-2, and GAD-7 scores. Respondents declaring their financial status as average differed significantly from those declaring their financial status as good in terms of: STAI X-1, STAI X-2, SHAI, and GAD-7 scores. Conclusions: The subjective experience of anxiety symptoms associated with fear of contracting COVID-19 was increased due to the ongoing pandemic, but was not significantly high in the analysed population of older people. COVID-19-related anxiety was significantly more common in lonely individuals and in those of worse financial status. Women and men differed significantly in terms of perceived state anxiety and trait anxiety measured by STAI. More studies addressing COVID-19-related anxiety in older people participating in the Polish Universities of the Third Age are needed to determine a more accurate distribution of this phenomenon in Poland.

## 1. Introduction

A series of cases of pneumonia of an unknown cause appeared at the end of 2019 in the Chinese province of Wuhan [1]. Several weeks later, in January 2020, the analysis of lab samples revealed a new virus, SARS-CoV-2, that induces an acute respiratory disease [2] which, on 11 February 2020, was referred to as COVID-19 by the World Health Organisation (WHO) Director-General, while on 11 March 2020, the WHO announced the pandemic [3]. On 27 July 2021, the number of all identified coronavirus infections in the world reached 194,995,684, of which 4,173,104 patients died due to the infection [4].

Fear of infection with SARS-CoV-2 has become widespread. It is constantly being enhanced by the media reports, where particular emphasis is put on the epidemiological data on mortality and incidence, dramatic information from intensive care units, and news about lacking healthcare resources. An additional factor that increases perceived anxiety is the so-called social distancing principle, which entails restrictions in interpersonal contact [5,6,7]. The estimated overall prevalence of anxiety in the general population during the COVID-19 pandemic is 25% [8]. In specific groups, the prevalence of fear of COVID-19 is varied, for example among medical students [9] and healthcare professionals [10] is also 25%, but among college students—36% [11].

All over the world, since the very beginning of the pandemic, older adults have been considered one of the groups at highest risk of SARS-CoV-2 infection and death due to COVID-19 [12]. The increased risk of contracting the disease and death depends on several factors. Firstly, Poland is one of the top European countries in terms of the number of confirmed COVID-19 cases. As of 27 July 2021, there have been 2,882,327 cases, of which 75,249 were fatal [13]. Moreover, the prevalence of chronic diseases, entailing multimorbidity, is high amongst the older adults all over the world, including in Poland, which increases the risk of severe health-related sequelae, including those related to COVID-19 [14]. The prevalence of multimorbidity in Poland is 38.7% [15].

Although, in principle, COVID-19 may evoke considerable fear among the older adults, there are no sufficient data about the perceived fear resulting from the ongoing COVID-19 pandemic in Poland, especially amongst actively ageing older people. The aim of the study was therefore to evaluate the severity of anxiety symptoms related to COVID-19 among the older adults who are participants of the Universities of the Third Age in Poland. In addition, it was evaluated how selected sociodemographic characteristics affect the level of anxiety symptoms in this subgroup of the older population from Poland. It was assumed that the level of anxiety symptoms among participants of the University of the Third Age will be relatively high. Additionally, we hypothesized that anxiety disorders related to the fear of COVID-19 would occur more frequently among women, single people, and people with lower education.

## 2. Materials and Methods

### 2.1. Participants

The study included participants of the University of the Third Age programme in Poland. A total of 296 persons were enrolled, including 258 women and 38 men. Over half of the respondents (54.73%) were in the age range from 60 to 69 years. A similar percentage of the respondents (51.35%) were married. Almost three-quarters of the University of the Third Age participants (71.96%) had higher education (Master’s degree). Nearly one-third of the older adults lived in large cities (over 500,000 inhabitants). The vast majority of the respondents (91.55%) were retired. Eighteen respondents had a history of mental disorders. These were: depressive disorders (*n* = 13), bipolar disorder (*n* = 1) and anxiety disorder (*n* = 4).

Detailed sociodemographic characteristics of the respondents are shown in Table 1.

### 2.2. Study Design and Data Collection

The cross-sectional study was conducted between 1 March and 10 May 2021. The invitations were sent to official e-mail addresses published on the websites of all University of the Third Age chapters in Poland. In this study, the snowball sampling method was employed; it is a non-random sample selection method consisting in recruiting participants by other participants (in our study by the Presidents of Universities of the Third Age or substantive coordinators the Universities that offer such forms of education for the older adults). We used this method in our study as we did not have a contact database for direct students of Universities of the Third Age.

The study involved an online survey created using dedicated software (Webankieta) (Get Feedback, Warsaw, Poland). A link to the survey was included in the invitation e-mails sent to the addresses available on the official University websites. The responses were recorded on the platform, and then downloaded as raw data prepared for a statistical analysis. The mean time to complete the survey was 29 min.

Apart from age and enrolment in the University of the Third Age programme, an additional inclusion criterion was written informed consent to participate in the study. The exclusion criteria were: (1) age ≤ 60 years; (2) illiteracy (a respondent who had completed at least primary school could participate in the study); and (3) lack of written consent to participate in the study. Each participant could withdraw from the study at any time.

The respondents were chosen by nonprobability sampling. Taking into account the total number of returned questionnaires, the rate of complete filling was 52.7%. The remaining questionnaires were incomplete (47.3%).

### 2.3. Measures

The study was a diagnostic survey, conducted with the use of the following validated psychometric scales: General Anxiety Disorder-7 (GAD-7), Short Health Anxiety Inventory (SHAI), and State-Trait Anxiety Inventory (STAI). The authors of the study asked respondents that questions from all standardized research tools used in the study should relate directly to fear of COVID-19.

#### 2.3.1. State-Trait Anxiety Inventory (STAI)

The structure of the STAI tool is based on differentiating between anxiety understood as a transitory and situation-dependent state, and anxiety understood as a relatively constant personality trait. In his concept, Spielberger referred to other, earlier studies conducted by R.B. Cattell and I.H. Scheier [16]. They identified two different factors. The first, responsible for situation-dependent score variability, was called “state anxiety”. The other, responsible for inter-individual differences, was termed “trait anxiety”. The STAI questionnaire consists of two independent parts, each containing 20 statements. The first part, STAI X-1, can evaluate anxiety considered as the current emotional state. This part of the questionnaire is a very sensitive tool. It enables one to trace the dynamics of anxiety even in short time intervals. The other part, STAI X-2, concerns anxiety understood as a personality trait [17]. The patient responds to each statement by choosing one of 4 options. The level of anxiety is expressed as the score resulting from summing up the sub-scores obtained for all individual responses. The scores from each part of the questionnaire may vary from 20 to 80. The test is interpreted in the following way: the higher the score, the higher the level of perceived anxiety. The score range of 39–40 in state anxiety evaluation suggests the detection of clinical symptoms of anxiety disorder [18,19]. The psychometric value of the test makes it useful in group studies. The test reliability, based on the internal consistency coefficient, varies from 0.76 to 0.92 in adult women and men. The construct validity of the X-1 scale ranges from 0.51 for men to 0.57 for women [17].

#### 2.3.2. Short Health Anxiety Inventory (SHAI)

The Short Health Anxiety Inventory (SHAI) is an 18-item scale designed to evaluate anxiety in two components of hypochondriasis: probability of disease and negative disease consequences. Each of the 18 items contains four statements of which respondents choose one that best describes their feelings over the past 6 months. The responses are scored on a 4-point scale where: 0—no symptoms, 1—mild symptoms, 2—severe symptoms, and 3—very severe symptoms. A cut-off score of 20 was optimal for detecting a severe form of health anxiety, providing the best balance between specificity and sensitivity [20,21].

#### 2.3.3. Generalised Anxiety Disorder Assessment (GAD-7)

The GAD-7 is a 7-item scale that is based on the Likert scale. It can be used when evaluating the level of anxiety and the risk of generalised anxiety disorder (GAD). The questions included in the survey evaluate the respondent’s feeling of anxiety, tension, nervousness, ability to control these feelings, how easily they appear and how problematic it is to relax. The respondent can obtain 0–3 points for each item, depending on the selected response, i.e., the frequency with which each problem occurs in the respondent (0—not at all; 1—several days; 2—more than half the days; 3—nearly every day). The evaluation concerns the past 14 days. The scores of 5, 10, and 15 indicate mild, moderate, and severe anxiety, respectively. The score of at least 10 indicates that generalised anxiety disorder is very likely [22].

### 2.4. Procedure and Ethical Considerations

The study was carried out in accordance with the recommendations, and was reviewed and approved by the Ethics Committee of the Medical University of Bialystok (No. R-I-002/592/2019). All subjects gave the written informed consent in accordance with the Declaration of Helsinki.

### 2.5. Statistical Analysis

The data were processed with Microsoft Excel 2020 spreadsheet and analysed with Statistica Data Miner C QC PL package. Normal distribution of quantitative variables was checked with the Shapiro–Wilk W-test. As none of the variables was distributed normally, they were analysed using non-parametric tests; the significance of the differences between two groups was verified with the Mann–Whitney U-test, and multiple groups were compared using Kruskal–Wallis ANOVA and appropriate post hoc tests. The associations between the pairs of quantitative variables were analysed based on the Spearman’s coefficients of rank correlation. The results of all tests were considered significant at *p* < 0.05.

## 3. Results

Table 2 presents descriptive statistics of the standardised research tools applied in this study. In two scales (STAI and SHAI), the mean scores demonstrated mild symptoms indicative of anxiety disorders in the older respondents. In the remaining scale (GAD-7), the means scores were not sufficient to identify the presence of anxiety symptoms related to COVID-19. The detailed values for individual variables are listed in Table 2.

Table 3 presents the prevalence of anxiety symptoms among students of University of the Third Age according to the standardized cut-off points. Most of the respondents showed anxiety symptoms measured by the STAI. Similarly, most older adults showed minimal or mild anxiety symptoms based on the GAD-7 scores. On the other hand, over 3/4 of the respondents did not show symptoms of anxiety disorders on the basis of the SHAI (Table 3).

The analysis of the scores obtained using the applied scales in the study group, taking into account the individual sociodemographic variables, indicated that women and men did differ significantly in terms of the scores obtained in STAI X-1 (*p* = 0.002) and STAI X-2 (*p* = 0.020) (Table 4).

There were no statistically significant differences between respondents with higher education and those with a different level of education. Nor were there any statistically significant differences between professionally active respondents and pensioners. A number of statistically significant differences were noted for the results of the applied scales in terms of the marital status. The analysis revealed that single respondents differed significantly from divorced ones in terms of STAI X-1 scores (*p* = 0.046). Moreover, widows/widowers differed significantly from divorced ones in terms of STAI X-2 (*p* = 0.045), and GAD-7 scores (*p* = 0.032) (Table 5).

Respondents declaring their financial status as average differed significantly from those declaring their financial status as good in terms of: STAI X-1, STAI X-2, SHAI, and GAD-7 scores. Details are included in Table 6.

When analysing the obtained data, no statistically significant association was found between age and scores of the individual scales. Moreover, the respondents were divided into subgroups by sex, marital status, level of education, and place of residence, and the associations between age and scale scores were also analysed within these individual subgroups. Taking into account the division by education, the significance was found for the correlation between age and STAI X-1 (r = −0.140, *p* = 0.042) in the group of people with higher education. As for the remaining divisions, the results obtained in the subgroups confirmed the results obtained in the full set analysis and, consequently, no significant correlation between age and scores of the applied scales was found.

The study also involved relationships between the values of all applied scales. It was found that each of the correlation coefficients was positive, high, and statistically significant (Table 7). Moreover, an analogous analysis was performed in subgroups, taking into account sex, marital status, education, and place of residence. All of the resultant correlation coefficients were positive, high, and statistically significant.

## 4. Discussion

This study is one of the first to determine the correlation between various sociodemographic factors and perceived anxiety related to the ongoing COVID-19 pandemic in the older Polish population. It was shown that the actively ageing older people experience various levels of anxiety symptoms related to COVID-19, depending on the applied scales. However, none of the scales yielded sufficiently high results to identify high COVID-19-related anxiety, and the set research hypothesis was therefore not confirmed in this study.

Although the present study demonstrated only mild anxiety symptoms in STAI and SHAI scales, other studies among the older adults from other countries have revealed that COVID-19 contributes to the worsening of mental health in this age group, primarily due to anxiety related to death, which is a consequence of this disease [23,24].

In our study, the prevalence of anxiety symptoms as measured by GAD-7 was 41.1%, including 28% of mild, 7.7% of moderate, and 5.4% of severe anxiety symptoms. Another Polish study, conducted using the same tool among university students, found that the prevalence of anxiety was high and was 65%, including 32% of mild, 21% of moderate, and 14% cases of severe anxiety disorder [25]. It is worth noting that higher percentages were recorded among young adults, not seniors. These differences could have been influenced by the timing of the study (young people were examined at the beginning of the pandemic) and the size of the group (the group of students was much larger than the group of seniors).

The present study showed that age was not linked significantly with anxiety related to developing COVID-19. These results are in line with previous studies, both Polish [26] and conducted abroad [27,28,29], which have shown that age is negatively correlated with anxiety symptoms during the pandemic. The available literature contains publications that did confirm high anxiety in the oldest age groups, particularly in China [30,31].

The present study did not show any statistically significant differences between professionally active respondents and those that finished their career (receiving retirement or disability pensions). The reverse was observed by Mistry et al. [32]. These authors found that older people who were unemployed or retired felt anxious about contracting COVID-19, and about its negative consequences, significantly more often. The same study [32] also confirmed a similar relationship as in the present study, namely that subjects with a worse financial status experienced stronger COVID-19-related anxiety than people with a better financial status.

The present study demonstrated that women were characterised by a higher level of COVID-19-related anxiety than men. The obtained values are in line with evidence provided by other authors confirming that women more often complain about stronger anxiety symptoms compared to men [25,33,34]. Other authors have also observed this relationship in their studies; for instance, in the study involving the Chinese population, women reported stronger worries related to developing COVID-19 than men [35]. In the present study, the overrepresentation of women compared to men in the study population could have affected the resulting higher level of anxiety. The finding that female sex is a predictor of anxiety suggests (as does the report of Naharci et al. [36]) that older women are more susceptible to mental health risks associated with COVID-19. The previous COVID-19-related studies reported mixed results regarding the association between sex and anxiety disorders. Two population-based studies showed that women tend to worry more often and are more susceptible to mental problems [37,38]. A recent literature review suggested that women have a tendency to develop a more psychological anxiety experience [39]. Nonetheless, further studies are needed to reflect sex-based differences in stress exposure related to developing infectious diseases.

In the study conducted by Islam et al. [40], it was shown that generalised anxiety was not significantly correlated with respondents’ education—a finding in line with the present study. Regarding educational level, the results revealed that older adults with higher education levels had lower levels of anxiety. This is consistent with the findings reported in Italy [41] and Egypt [42] which showed that lower level of education directly indicates the beginning of the increased pandemic stress. This may be attributed to the fact that education is the foundation for successful coping. On the other hand, some studies suggested that people with higher education tended to be more concerned, perhaps due to a high degree of self-awareness of their well-being [30]. The novel nature of coronavirus disease and changing perceptions about the disease are one of the main factors justifying these opposite results.

The current facts suggest that certain groups of people (for example, older adults) may be especially prone to experiencing generalised anxiety in the initial stages of the COVID-19 pandemic, and further investigations are needed to specify the causes and develop appropriate interventions to improve the public health of the entire population.

### Limitations

The present study does have certain, potential limitations. First, it was a cross-sectional study, based only on a self-reportable survey. Although the applied scales are sensitive tools designed to detect anxiety symptoms, they all focus on subjective symptoms, rather than objective clinical criteria, which poses a risk of false-positive results. Second, the study group was too small to generalise the results to the whole population of Polish older people attending Universities of the Third Age. Third, the study group was overrepresented by women, and therefore the results should be also verified in a larger group of men. However, both the actual rate of University of the Third Age participants and the actual demographic trends in the Polish population distinguish a high rate of women in relation to men. Despite these limitations, the results of this research can be a starting point for further studies addressing anxiety symptoms caused by fear of developing COVID-19 and its sociodemographic determinants amongst participants of Universities of the Third Age. A longitudinal study could provide optimal answers to these questions.

## 5. Conclusions

1. The subjective experience of anxiety symptoms associated with fear of contracting COVID-19 was increased due to the ongoing pandemic, but was not significantly high in the analysed population of older people;
2. COVID-19-related anxiety was significantly more common in lonely individuals (widows/widowers, divorced individuals, and single persons) and in those of worse financial status;
3. Women and men differed significantly in terms of perceived state anxiety and trait anxiety measured by STAI;
4. More studies are needed addressing COVID-19-related anxiety in the older adults participating in the Polish Universities of the Third Age to determine a more accurate distribution of this phenomenon in Poland.

## Figures and Tables

**Table 1:** Sociodemographic characteristics of the respondents.

Sociodemographic Feature		*n*	%
Sex	male	38	87.16%
	female	258	12.84%
Age	60–69 years	162	54.73%
	70–79 years	118	39.86%
	80–89 years	16	5.41%
Marital status	married	152	51.35%
	widow/widower	75	25.34%
	separated	20	6.76%
	divorced	46	15.54%
	single	3	1.01%
Education	higher	213	71.96%
	secondary	76	25.68%
	technical	6	2.03%
	vocational	1	0.34%
Place of residence	village	24	8.11%
	town up to 50 thousand	84	28.38%
	town up to 200 thousand	37	12.50%
	city up to 500 thousand	48	16.22%
	city over 500 thousand	103	34.80%
Financial status	very bad	1	0.34%
	bad	2	0.68%
	average	114	38.51%
	good	153	51.69%
	very good	26	8.78%
Socio-professional status	retirement pensions	271	91.55%
	disability pensions	4	1.35%
	professionally active	21	7.09%
Voivodeship	Lower Silesian	32	10.81%
	Kuyavian-Pomeranian	2	0.68%
	Lublin	12	4.05%
	Lubusz	7	2.36%
	Łódź	1	0.34%
	Lesser Poland	67	22.64%
	Masovian	49	16.55%
	Opole	2	0.68%
	Subcarpathian	7	2.36%
	Podlaskie	61	20.61%
	Pomeranian	9	3.04%
	Silesian	23	7.77%
	Świętokrzyskie	4	1.35%
	Warmian-Masurian	16	5.41%
	Greater Poland	1	0.34%
	West Pomeranian	3	1.01%
History of mental disorders	yes	18	6.08%
	no	278	93.92%

**Table 2:** Descriptive statistics for the scales applied in the study.

	$\bar{x}$	SD	Min.	Q_1_	Me	Q_3_	Max.
STAI X-1	44.48	11.38	20.00	37.00	43.00	53.00	77.00
STAI X-2	41.83	8.59	21.00	36.00	41.50	47.00	67.00
SHAI	14.62	7.23	1.00	9.00	14.00	19.00	41.00
GAD-7	4.51	4.66	0.00	1.00	3.00	7.00	21.00

Abbreviations: GAD-7—General Anxiety Disorder-7, Max.—maximum, Me—median, Min.—minimum, SD—standard deviation, SHAI—Short Health Anxiety Inventory, STAI—State-Trait Anxiety Inventory, Q_1_—lower quartile, Q_3_—upper quartile, and $\bar{x}$—mean.

**Table 3:** Prevalence of anxiety symptoms among students of University of the Third Age according to the standardized cut-off points.

	Cut-Off Points	*n*	%
STAI X-1	<40 (no symptoms)	103	34.7%
	≥40 (anxiety symptoms)	194	65.3%
STAI X-2	<40 (no symptoms)	119	40.1%
	≥40 (anxiety symptoms)	178	59.9%
SHAI	<20 (no symptoms)	226	76.1%
	≥20 (anxiety symptoms)	71	23.9%
GAD-7	0 (no symptoms)	66	22.2%
	1–4 (minimal symptoms)	109	36.7%
	5–9 (mild symptoms)	83	28.0%
	10–14 (moderate symptoms)	23	7.7%
	15–21 (severe symptoms)	16	5.4%

Abbreviations: GAD-7—General Anxiety Disorder-7, SHAI—Short Health Anxiety Inventory, and STAI—State-Trait Anxiety Inventory.

**Table 4:** Impact of sex on the scores obtained in the psychometric scales applied in the study.

	Women (*n* = 258)		Men (*n* = 38)		*p*
	$\bar{x}\pm SD$	Me	$\bar{x}\pm SD$	Me	
STAI X-1	45.26 ± 11.56	44	39.21 ± 8.39	39	0.002 *
STAI X-2	42.27 ± 8.73	42	38.84 ± 6.88	38	0.020 *
SHAI	14.84 ± 7.39	14	13.08 ± 5.92	12	0.205
GAD-7	4.69 ± 4.82	4	3.26 ± 3.23	2	0.195

Abbreviations: GAD-7—General Anxiety Disorder-7, Me—median, *p*—*p*-value, SD—standard deviation, SHAI—Short Health Anxiety Inventory, STAI—State-Trait Anxiety Inventory, $\bar{x}$—mean, and *—statistically significant.

**Table 5:** Impact of marital status on the scores obtained in the psychometric scales applied in the study *.

	Married (I) (*n* = 152)		Widow/Widower (II) (*n* = 75)		Separated (III) (*n* = 20)		Divorced (IV) (*n* = 46)		*p*	
	$\bar{x}\pm SD$	Me	$\bar{x}\pm SD$	Me	$\bar{x}\pm SD$	Me	$\bar{x}\pm SD$	Me	K-W	Post hoc
STAI X-1	44.68 ± 10.91	43	43.07 ± 11.44	42	39.8 ± 8.41	40.5	48.59 ± 12.91	48	0.046	III–IV: 0.046
STAI X-2	41.89 ± 7.54	42	40.41 ± 8.84	40	38.8 ± 8.68	39	45.39 ± 10.51	46	0.021	II–IV: 0.045
SHAI	14.01 ± 6.89	13	14.37 ± 7.12	13	14.6 ± 6.75	15	17.04 ± 8.5	16.5	0.1636	-
GAD-7	4.72 ± 4.64	4	3.41 ± 3.87	3	3.45 ± 3.9	2	6.24 ± 5.75	4.5	0.017	II–IV: 0.032

* The table also includes statistically significant results of the post hoc tests; for the remaining comparisons *p* > 0.05. Abbreviations: GAD-7—General Anxiety Disorder-7, K-W—Kruskal–Wallis test, Me—median, *p*—*p*-value, SD—standard deviation, SHAI—Short Health Anxiety Inventory, STAI—State-Trait Anxiety Inventory, and $\bar{x}$—mean.

**Table 6:** Impact of financial status on the scores obtained in the psychometric scales applied in the study *.

	Very Good (I) (*n* = 26)		Good (II) (*n* = 153)		Average (III) (*n* = 114)		*p*	
	$\bar{x}\pm SD$	Me	$\bar{x}\pm SD$	Me	$\bar{x}\pm SD$	Me	K-W	Post hoc
STAI X-1	43 ± 11.85	41	42.84 ± 10.65	41	46.72 ± 11.83	47	0.010	II–III: 0.011
STAI X-2	39.5 ± 9.58	39	40.56 ± 7.68	40	43.82 ± 8.93	45	0.002	II–III: 0.005
SHAI	12.92 ± 7.21	11.5	13.71 ± 6.66	13	16.03 ± 7.49	15	0.017	II–III: 0.035
GAD-7	3.96 ± 4.46	3	3.85 ± 4.25	3	5.39 ± 5.01	4	0.023	II–III: 0.027

* The table also includes statistically significant results of the post hoc tests; for the remaining comparisons *p* > 0.05. Abbreviations: GAD-7—General Anxiety Disorder-7, K-W—Kruskal–Wallis test, Me—median, *p*—*p*-value, SD—standard deviation, SHAI—Short Health Anxiety Inventory, STAI—State-Trait Anxiety Inventory, and $\bar{x}$—mean.

**Table 7:** Spearman’s rank correlations between standardised psychometric scales used in the study.

		STAI X-1	STAI X-2	SHAI
STAI X-2	r	0.789	---	---
	*p*	<0.001 *		
SHAI	r	0.584	0.625	---
	*p*	<0.001 *	<0.001 *	
GAD-7	r	0.691	0.758	0.554
	*p*	<0.001 *	<0.001 *	<0.001 *

Abbreviations: GAD-7—General Anxiety Disorder-7, *p*—*p*-value, r—Spearman’s rank correlation coefficient, SHAI—Short Health Anxiety Inventory, STAI—State-Trait Anxiety Inventory, and *—statistically significant.

## Data Availability

Data are available upon reasonable request.
